# Clinical Experience With a High-Definition Three-Dimensional Exoscope for Spinal Surgery: A Review of 30 Consecutive Initial Cases

**DOI:** 10.7759/cureus.116402

**Published:** 2026-09-17

**Authors:** Kimiaki Sato, Kimiaki Yokosuka, Tsunemasa Matsubara, Suguto Futami, Mizuki Kakizoe, Masafumi Goto, Kei Yamada, Koji Hiraoka

**Affiliations:** 1 Department of Orthopaedic Surgery, Kurume University School of Medicine, Kurume, JPN

**Keywords:** cervical compressive myelopathy, clinical experience, exoscope, initial cases, lumbar degenerative disease, spinal surgery

## Abstract

Background: The use of exoscopes with high-definition three-dimensional displays has become increasingly common; however, their use in spinal surgery remains limited. We retrospectively evaluated initial cases of spinal surgery performed using an exoscope. Materials and methods: Between May 2019 and May 2023, we performed 30 consecutive spinal surgeries using the exoscope, including 22 lumbar decompression surgeries and eight cervical laminoplasties. We evaluated clinical outcomes using the Japanese Orthopedic Association (JOA) score, the recovery rate calculated from the JOA score, and patient self-evaluation. Results: All procedures were completed using the exoscope without switching to other visualization devices. The outcomes were satisfactory, and no patients showed postoperative worsening of the JOA score. No exoscope-related surgical complications were observed. Conclusion: This novel exoscope system demonstrated feasibility and acceptable safety in the initial cases.

## Introduction

An exoscope is a visualization device first reported by Gildenberg in the 1990s [1,2]. The exoscope, which combines features of both the surgical microscope and endoscope, has become increasingly popular in recent years [3,4]; however, its use in spinal surgery remains limited. One such exoscope, ORBEYE™ (OLYMPUS, Tokyo, Japan), released in 2017 [5], offers four times the resolution of standard high-definition imaging (4K resolution) and high-quality, three-dimensional (3D) imaging. Our institution has used this exoscope since August 2020. We published our preliminary experience with the exoscope in 11 cases of spinal disorders in 2022 [3]. Since then, we have gained further experience. In this study, we reviewed data from our initial 30 cases, including one case performed before we introduced this device at our institution. This retrospective observational study evaluates the early learning experience with the exoscope.

## Materials and methods

The inclusion criteria for this study were (1) patients and their families who provided informed consent for spinal surgery, (2) patients with lumbar degenerative disorders who were candidates for posterior decompression surgery, (3) patients with cervical compressive myelopathy who were candidates for laminoplasty, and (4) patients for whom approval for exoscopic surgery was obtained from the operating room. No cases meeting the inclusion criteria were excluded, including those with missing follow-up data. The Institutional Review Board of Kurume University approved the study (No. 25169).

Between May 2019 and May 2023, we performed 30 consecutive spinal surgeries using this exoscope (Table 1), including 22 lumbar decompression surgeries and eight cervical laminoplasties. One surgeon performed all 30 cases, with assistance from 13 residents and instructors. The primary surgeon, who had no prior experience with this exoscope, was a board-certified spine surgeon approved by the Board of the Japanese Society for Spine Surgery and Related Research (JSSR), qualified by the Japanese Society of Endoscopic Surgery (JSES) and the Japanese Orthopedic Association (JOA) to perform endoscopic spinal surgery, and had over 25 years of experience as a primary operator. The 13 residents and instructors also had no prior experience or training with this exoscope.

**Table 1:** Patient characteristics #1 For the cervical disorder group, the values indicate the number of expanded laminae; for the lumbar disorder group, they indicate the number of decompressed intervertebral levels. All patients in the cervical disorders group underwent additional distal and/or proximal dome-like laminoplasty. Moreover, two-level cervical foraminotomy was performed in one patient with CSMR. ASA-PS: American Society of Anesthesiologists Physical Status Classification System, BMI: body mass index, CSM: cervical spondylotic myelopathy, CSMR: cervical spondylotic myeloradiculopathy, DS: degenerative spondylolisthesis, LSS: lumbar spinal stenosis, OPLL: ossification of posterior longitudinal ligament, re-op: re-operation, SPSL: spinous process-splitting laminectomy

	Lumbar disorders (n = 22)	Cervical disorders (n = 8)
Age (year)	72.8 ± 5.7 (63-81)	63.0±10.7 (45-79)
Gender (male/female)	12/10	7/1
BMI (kg/m^2^)	24.0 ± 3.0 (18.3-29.0)	26.6 ± 5.5 (19.1-36.9)
ASA-PS (1/2/3)	2/19/1	1/6/1
Diagnosis	LSS: 10	OPLL: 5
	LSS (DS): 7	CSM: 2
	LSS (re-op): 5	CSMR: 1
Surgical procedure	SPSL: 14	Double-door laminoplasty: 7
	Conventional laminectomy: 8	Open-door laminoplasty: 1
Decompression level^ #1^	2.2 ± 0.5 (1-3)	5.3 ± 0.7 (4-6)

Figure 1 shows the basic exoscope setup for lumbar spinal surgery in the operating room. We performed all 30 cases using a dual-monitor system. Two monitors were arranged so that the primary surgeon and the assistant surgeon did not obstruct each other’s view. The surgeons wore polarized 3D glasses to view the images correctly (Figure 2). Before surgery, we generally set the monitor to the 3D display mode and switched to the 2D display mode as requested.

**Figure 1:**
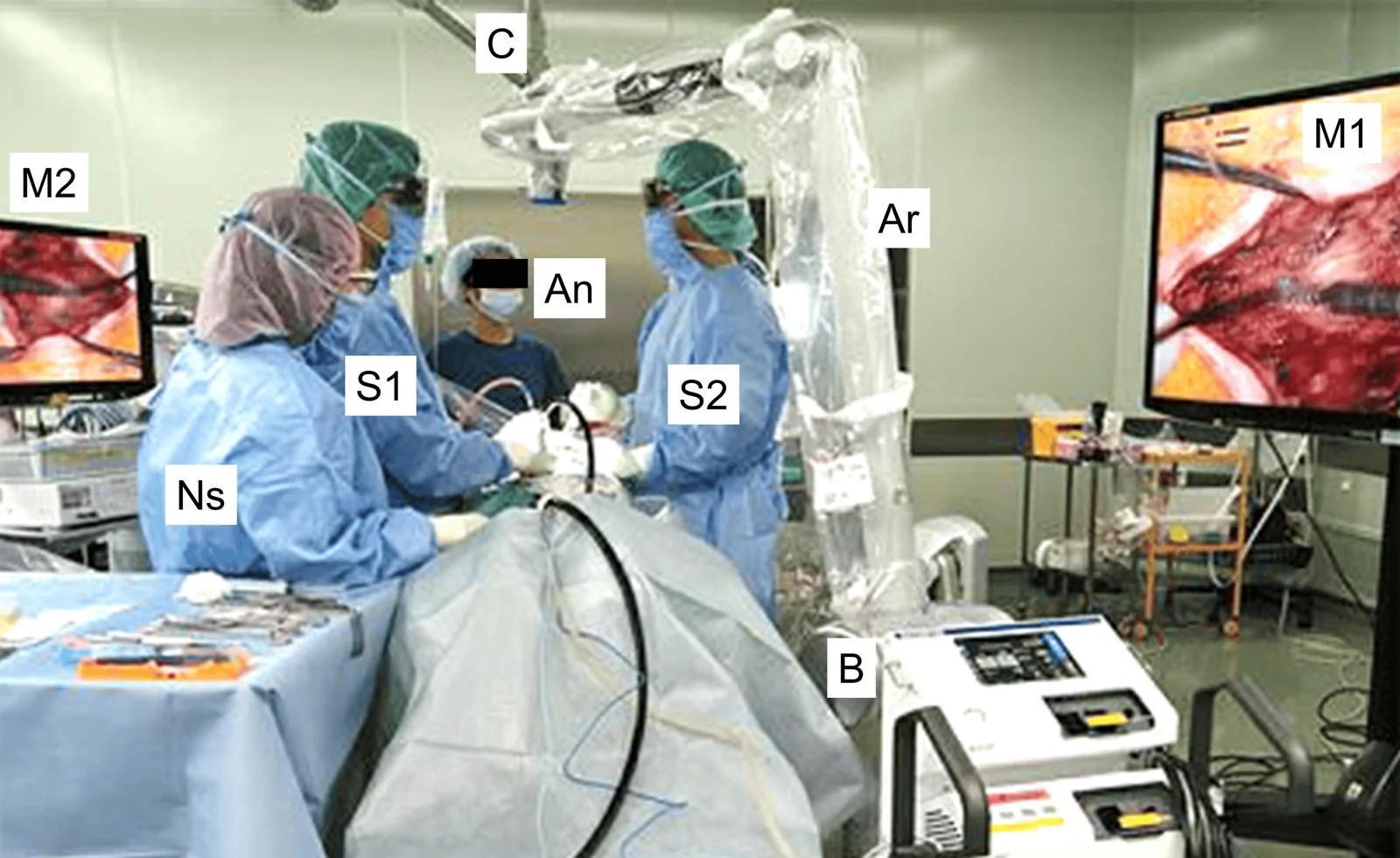
Dual-monitor setup for lumbar spinal surgery showing the body of the exoscope placed on the left side of the assistant surgeon caudal to the patient S1: main surgeon, S2: assistant surgeon, An: anesthesiologist, Ns: nurse, M1: 55-inch main monitor, M2: 31-inch secondary monitor, Ar: arm, B: body, C: camera

**Figure 2:**
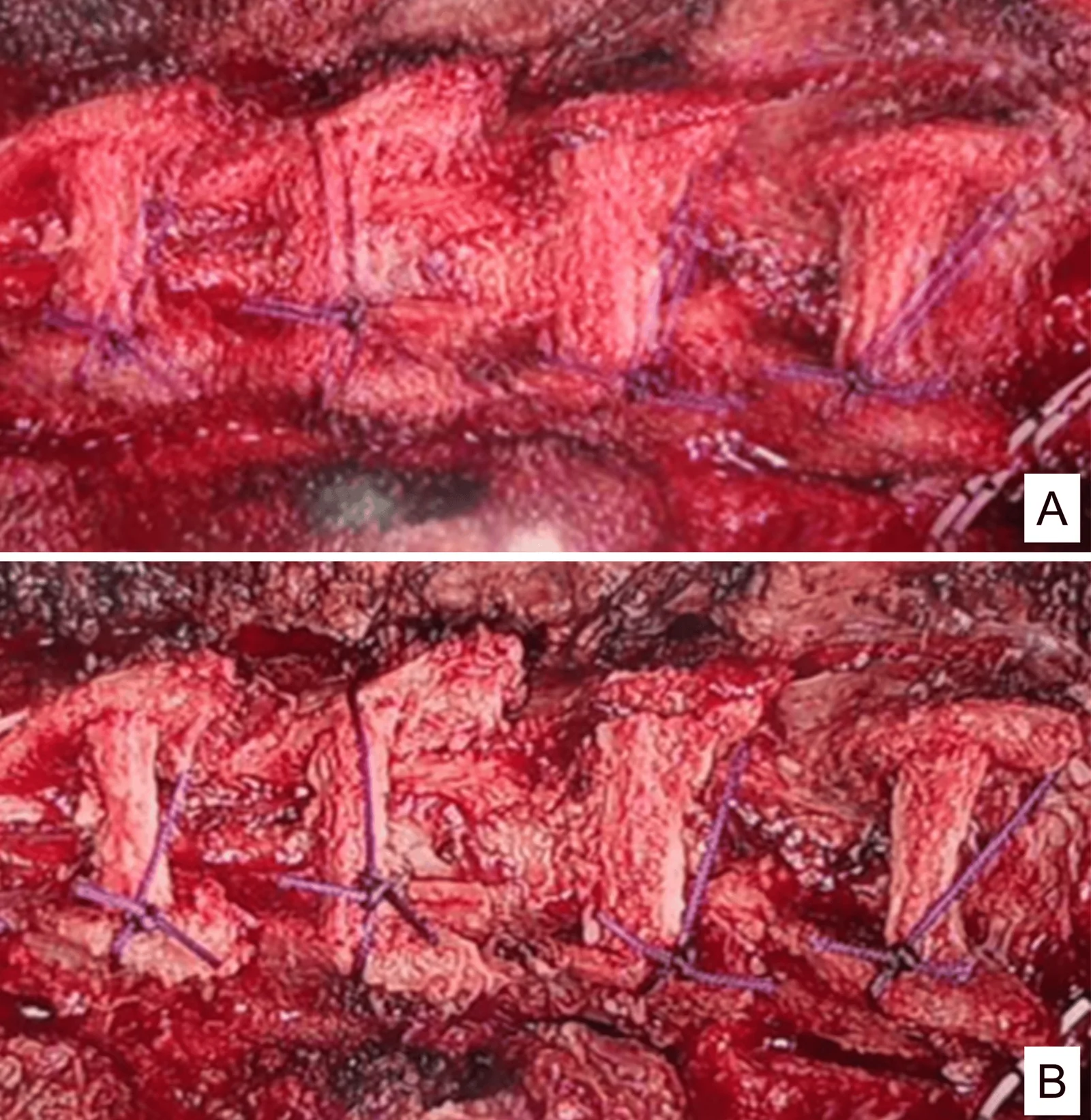
Cervical laminoplasty A double-door laminoplasty from C4 to C7 has been performed (rostrally to the left and caudally to the right). The spinous process was partly resected, and the removed spinous process was implanted between the expanded laminae. The 3D screen appears blurred to the naked eye (A). However, when viewed through 3D glasses, the image appears 3D. The exoscope can also display 2D images (B). 2D: two-dimensional, 3D: three-dimensional

Postoperative management involved permitting walking a few days after surgery. We used a soft brace for three months in patients with lumbar disorders and a Philadelphia collar for three weeks in patients with cervical disorders after surgery.

We evaluated clinical results using the JOA scoring system for low back pain [6] and cervical myelopathy (17-2 version) [7] at the final follow-up. We calculated the recovery rate using a formula [8]. For subjective evaluation, we also collected patients' self-assessments and categorized the ratings into four levels.

Statistical analysis

We used EZR (Saitama Medical Center, Jichi Medical University, Saitama, Japan) for statistical analysis [9]. We reviewed all clinical data retrospectively and presented them as mean ± standard deviation (SD) with range, or as counts. Differences between preoperative and postoperative JOA scores were analyzed using the Wilcoxon signed-rank test. A p-value of < 0.05 was considered statistically significant.

## Results

All procedures were completed with this exoscope, and no case required switching to other visualization devices. The mean follow-up period was 21.4 ± 12.4 months (range 6-56) for all 30 cases, and no patients showed a postoperative decrease in the JOA score. The recovery rate was 64.7 ± 24.1% (range 30-100), and the subjective evaluations were as follows: (a) completely relieved in 6 patients, (b) better in 21 patients, (c) unchanged in three patients, and (d) worse in 0 patients among all 30 cases. Table 2 shows the clinical outcomes for lumbar and cervical disorders.

**Table 2:** Clinical outcomes The JOA score for lumbar and cervical disorders was significantly improved postoperatively compared with preoperatively (Wilcoxon signed-rank test, W = 0, *p < 0.05, ***p < 0.001). #2 Recovery rate = (postoperative JOA score − preoperative JOA score) / (normal JOA score − preoperative JOA score) × 100. #3 Patient self-evaluation (a: completely relieved, b: better, c: unchanged, d: worse). JOA: Japanese Orthopedic Association

		Lumbar disorders (n = 22)	Cervical disorders (n = 8)
Operative time (minutes)		138.5 ± 28.4 (85-202)	190.5 ± 37.8 (141-257)
Blood loss (g)		116.2 ± 110.9 (10-490)	132.9 ± 157.6 (22-512)
Preoperative JOA score		16.2 ± 2.4 (11-22)	10.0 ± 1.3 (8.0-12.0)
Postoperative JOA score		25.3 ± 3.1 (17-29)***	13.4 ± 1.4 (11.0-15.5)*
Recovery rate (%)^ #2^		70.4 ± 23.9 (14.3-100)	49.1 ± 17.6 (30-73.3)
Follow-up (months)		16.9 ± 8.7 (6-36)	33.9 ± 13.2 (15-56)
Patient self-evaluation^ #3^	a	6	0
	b	14	7
	c	2	1
	d	0	0

The JOA score for lumbar disorders was significantly improved postoperatively compared with preoperatively (Wilcoxon signed-rank test, W = 0, p = 0.0000419). The JOA score for cervical disorders also improved significantly postoperatively (Wilcoxon signed-rank test, W = 0, p = 0.0141). The recovery rate of the spinous process-splitting laminectomy (SPSL) method (n = 14) for lumbar disorders was 74.5 ± 26.0% (range 14.3-100%), and that of conventional laminectomy (n = 8) was 63.2 ± 19.0% (range 38.5-86.7%). No surgical complications, such as dural tear or neurovascular injury, were related to exoscope use in any of the 30 cases. No blood transfusions were required.

One patient with a cervical disorder developed a surgical site infection (SSI) after the surgery, which was controlled with surgical debridement. One patient with a lumbar disorder had residual symptoms after surgery and underwent selective nerve root blocks.

Two assistants experienced virtual reality sickness during surgery; these events occurred early in their experience with the exoscopic system. One had mild symptoms and completed the procedure; the symptoms improved with more experience using the exoscope. The other completed all five procedures by switching the monitor from 3D to 2D.

## Discussion

In the present study, we evaluated the clinical outcomes of 30 initial cases of exoscopic spinal surgery. We assessed outcomes using the JOA score, and none of the patients showed deterioration from preoperative levels. Additionally, JOA scores for both lumbar and cervical disorders improved significantly at the final follow-up. We observed no intraoperative complications, such as dural tear or neurovascular injury. Although all procedures in our study were completed using the exoscope, a recent study reported conversion from an exoscope to a traditional microscope in 3.1% of cases [10].

The exoscope's small, flexible camera allows surgeons to view the surgical field from various angles, enabling multiple surgical positions, approaches, and techniques without compromising the surgeon’s posture or causing discomfort [4,5]. Surgeons can maintain an ergonomic “head-up” posture, thereby reducing strain on the head and neck during surgery. Furthermore, a large monitor allows multiple staff members to view the same high-quality images as the surgeons. As mentioned earlier, using an exoscope in spinal surgery offers several advantages. However, eye strain and virtual reality sickness have been reported during surgeries using 3D monitors [11,12]. Although these symptoms may improve with experience, the ability to switch the display from 3D to 2D may help mitigate these issues.

Initially, we placed the exoscope body on the patient's cephalic side for both lumbar and cervical disorders in our institution. In 23 of the 30 cases in this study, the exoscope arm did not obstruct the view, and camera operation was also straightforward when the exoscope body was placed on the caudal side of the patient in lumbar disorders. Therefore, in this study, the body of the exoscope was placed on the cephalic side in 16 of 22 cases and on the caudal side in the remaining six cases for lumbar disorders.

Previously, we had used conventional laminectomy for lumbar decompression surgery; however, in May 2021, we switched to the SPSL method [13]. Therefore, in this study, we treated eight patients with lumbar disorders using the conventional method and 14 cases using the SPSL method.

In previous reports, the recovery rate of the SPSL method for lumbar disorders ranged from 60.1% to 75.0% with a follow-up period of 12.0 to 31.2 months [13-17], whereas that of conventional laminectomy ranged from 40.3 to 74.0% with a follow-up period of 12.0 to 38.7 months (Table 3) [13-16]. The surgical outcomes in this study, as assessed using the JOA score recovery rate, may be comparable to those previously reported for lumbar disorders [13-17]. However, further studies are needed before definitive conclusions can be drawn because this was a historical comparison without a control group.

**Table 3:** Recovery rate of SPSL and conventional laminectomy for lumbar disorders F/U: follow-up, RR: recovery rate, SPSL: spinous process-splitting laminectomy

Authors	SPSL		conventional laminectomy	
	RR (%)	F/U (months)	RR (%)	F/U (months)
Watanabe et al. (2005) [13]	67.6	29	59.2	38.7
Watanabe et al. (2011) [14]	75	12	74	12
Uehara et al. (2014) [15]	63.8	24	40.3	24
Kanbara et al. (2015) [16]	64.2	12	68.7	12
Maruo et al. (2015) [17]	60.1	31.2		
Range (min-max)	60.1-75.0	12.0-31.2	40.3-74.0	12.0-38.7

In contrast, recovery rates for laminoplasty for cervical spondylotic myelopathy (CSM) have been reported as 55-60% [18,19], and for ossification of the posterior longitudinal ligament (OPLL) as 45-62% [20]. The recovery rates observed in patients with cervical disorders were lower than those reported in previous studies [18-20]. In this study, six of the eight cases with cervical disorders had lumbar spinal stenosis (LSS), and three of them had previously undergone surgical treatment. One of the two patients without LSS had diffuse idiopathic skeletal hyperostosis. These factors may explain the relatively poor recovery rate in this study. The analysis of the small cervical subgroup (n = 8) is underpowered, and the proposed explanation should therefore be considered hypothesis-generating rather than confirmatory. Because this study included only early cases, further experience and longer follow-up are needed.

Complications occur at a high rate with new visualization devices, especially in the first 30 cases [21]. In minimally invasive spine surgery, one disadvantage has been the use of 2D imaging [21]. One possible reason why no major problems occurred during this period is that the surgeries were performed while viewing a large, high-resolution 4K 3D display.

Limitations

This clinical experience has several limitations. First, this was a single-center retrospective study of early cases with a small sample size, heterogeneous follow-up (6 to 56 months), and historical comparison without a control group. Second, exoscopic surgery was limited to cervical compressive myelopathy and lumbar degenerative disease, and surgical procedures were limited to cervical laminoplasty and lumbar decompression surgery. Third, our institution has only one exoscope, which several surgical departments share, including neurosurgery, otolaryngology, urology, and plastic surgery. When the exoscope was unavailable, we used conventional surgical microscopes for spinal surgeries. These factors may have introduced bias into the clinical results.

## Conclusions

We retrospectively evaluated our initial 30 consecutive spinal surgery cases performed using a high-definition 3D exoscope. All procedures were completed with the device, and outcomes were satisfactory, with no major complications. This study suggests that this novel exoscopic system is feasible and has an acceptable safety profile in these initial spinal surgery cases.

## References

[1] Gildenberg PL, Ledoux R, Cosman E, Labuz J (1994). The exoscope--a frame-based video/graphics system for intraoperative guidance of surgical resection. Stereotact Funct Neurosurg.

[2] Gildenberg PL, Labuz J (1997). Stereotactic craniotomy with the exoscope. Stereotact Funct Neurosurg.

[3] Sato K, Yokosuka K, Yoshida T (2023). Preliminary clinical experience with a high-definition three dimensional exoscope for spinal surgery. Kurume Med J.

[4] Innocenti N, Corradino N, Restelli F (2024). High-definition 4K-3D exoscope in spine surgery: a single-center experience and review of the literature. Medicina (Kaunas).

[5] (2026). ORBEYE™ exoscope: the future of surgery is looking up. http://medical.olympusamerica.com/products/orbeye.

[6] Fujiwara A, Kobayashi N, Saiki K, Kitagawa T, Tamai K, Saotome K (2003). Association of the Japanese Orthopaedic Association score with the Oswestry Disability Index, Roland-Morris Disability Questionnaire, and Short-Form 36. Spine (Phila Pa 1976).

[7] Yonenobu K, Abumi K, Nagata K, Taketomi E, Ueyama K (2001). Interobserver and intraobserver reliability of the japanese orthopaedic association scoring system for evaluation of cervical compression myelopathy. Spine (Phila Pa 1976).

[8] Hirabayashi K, Miyakawa J, Satomi K, Maruyama T, Wakano K (1981). Operative results and postoperative progression of ossification among patients with ossification of cervical posterior longitudinal ligament. Spine (Phila Pa 1976).

[9] Kanda Y (2013). Investigation of the freely available easy-to-use software 'EZR' for medical statistics. Bone Marrow Transplant.

[10] Begagić E, Pugonja R, Bečulić H, Selimović E, Skomorac R, Saß B, Pojskić M (2024). The new era of spinal surgery: exploring the use of exoscopes as a viable alternative to operative microscopes-a systematic review and meta-analysis. World Neurosurg.

[11] Chen Y, Wu Z (2023). A review on ergonomics evaluations of virtual reality. Work.

[12] Hashikata H, Maki Y, Hayashi H (2024). Assessment of head-mounted display for exoscopic neurosurgery. Surg Neurol Int.

[13] Watanabe K, Hosoya T, Shiraishi T, Matsumoto M, Chiba K, Toyama Y (2005). Lumbar spinous process-splitting laminectomy for lumbar canal stenosis. Technical note. J Neurosurg Spine.

[14] Watanabe K, Matsumoto M, Ikegami T (2011). Reduced postoperative wound pain after lumbar spinous process-splitting laminectomy for lumbar canal stenosis: a randomized controlled study. J Neurosurg Spine.

[15] Uehara M, Takahashi J, Hashidate H (2014). Comparison of spinous process-splitting laminectomy versus conventional laminectomy for lumbar spinal stenosis. Asian Spine J.

[16] Kanbara S, Yukawa Y, Ito K, Machino M, Kato F (2015). Surgical outcomes of modified lumbar spinous process-splitting laminectomy for lumbar spinal stenosis. J Neurosurg Spine.

[17] Maruo K, Tachibana T, Inoue S, Arizumi F, Yoshiya S (2015). Prognostic factors of surgical outcome after spinous process-splitting laminectomy for lumbar spinal stenosis. Asian Spine J.

[18] Watanabe M, Chikuda H, Fujiwara Y (2023). Japanese Orthopaedic Association (JOA) clinical practice guidelines on the management of cervical spondylotic myelopathy, 2020-secondary publication. J Orthop Sci.

[19] Matz PG, Anderson PA, Groff MW (2009). Cervical laminoplasty for the treatment of cervical degenerative myelopathy. J Neurosurg Spine.

[20] Kawaguchi Y, Imagama S, Iwasaki M (2021). Japanese Orthopaedic Association (JOA) clinical practice guidelines on the management of ossification of the spinal ligament, 2019. J Orthop Sci.

[21] Sharif S, Afsar A (2018). Learning curve and minimally invasive spine surgery. World Neurosurg.

